# Indigenous perspectives on wellness and health in Canada: study protocol for a scoping review

**DOI:** 10.1186/s13643-020-01428-0

**Published:** 2020-08-11

**Authors:** K. Thiessen, M. Haworth-Brockman, R. Stout, P. Moffitt, J. Gelowitz, J. Schneider, L. Demczuk

**Affiliations:** 1 College of Nursing, Rady Faculty of Health Sciences, University of Manitoba, 89 Curry Place, Winnipeg, Manitoba R3T 2N2 Canada; 2 Community Health Sciences, Rady Faculty of Health Sciences, University of Manitoba, 750 Bannatyne Avenue, Winnipeg, Manitoba R3E 0W3 Canada; 3 National Collaborating Centre for Indigenous Health, 3333 University Way, Prince George, British Columbia V2N 4Z9 Canada; 4 Aurora Research Institute/Aurora College, 5004 54th Street, Yellowknife, Northwest Territories X1A 2R3 Canada; 5 Max Rady Faculty of Medicine, Rady Faculty of Health Sciences, University of Manitoba, 583 Elgin Avenue, Winnipeg, Manitoba R3A 0L2 Canada; 6 University of Manitoba Libraries, University of Manitoba, 25 Chancellor’s Circle, Winnipeg, Manitoba R3T 0E7 Canada

**Keywords:** Scoping review, Indigenous, Wellness, Well-being, Canada

## Abstract

**Background:**

Indigenous communities are often portrayed from a deficit-based lens; however, Indigenous communities have self-determined perspectives of health and well-being that are strength based. The objective of this study will be to systematically map the literature on perspectives, concepts, and constructs of wellness and well-being in Indigenous communities in Canada.

**Methods:**

A scoping review protocol was designed following the Arksey and O’Malley framework. We will search the following electronic databases (from inception onwards): MEDLINE, EMBASE, Web of Science, CINAHL, Academic Search Complete, Anthropology Plus, Bibliography of Native North Americans, Canadian Business and Current Affairs, and Circumpolar Health Bibliographic Database. Grey literature will be identified through searching dissertation databases, Google Scholar, and conference abstracts. We will include all types of literature in English, published and unpublished, including any study design, reviews and meta-analyses, dissertations, reports, and books. The literature considered should describe or reflect Indigenous perspectives that identify concepts or constructs related to well-being or wellness; literature can be from any setting in Canada. Two reviewers will independently screen all citations, full-text reports, and abstract data. Data analysis will involve quantitative descriptions (e.g. frequencies) and qualitative content analysis methods.

**Discussion:**

This review will provide a synthesis of the literature on Indigenous perspectives, concepts, and constructs of wellness and well-being in Canada. We anticipate the study will contribute to improve our understanding of how Indigenous communities conceptualize and embody wellness. Our findings will provide a basis for engaging Indigenous stakeholders in future health research and informing future interpretations of how wellness is conceptualized, whether written or unwritten.

## Background

Western conceptualizations of health reflecting colonialist regimes portray Indigenous communities from a deficit-based lens while ignoring communities’ strengths and self-determined perspectives of health and well-being [1–3]. It is known that the biomedical model focuses on secondary prevention, which is the prevention of disease [4], thus disease focused. Furthermore, ongoing and historical colonization of Indigenous persons results in destructive mechanisms with significant detrimental consequences for the health and well-being of Indigenous communities [5, 6].

In the last decade, articles and reports in the literature have demonstrated that numerous studies have sought nation-specific Indigenous perspectives and constructs of wellness and well-being. For example, some articles identified constructs such as connection to culture (i.e. engaging in Indigenous philosophies, beliefs, practices, and values), community, and family and traditional activities as restorative to well-being [7–17]. Connection to land, nutrition, food, and financial security was also viewed as important to the wellness of a community [18–20], and specific articles discussed mental wellness from Indigenous youths’ perspectives [10, 21]. Indigenous indicators of wellness must be culturally sensitive, reflecting a positive view of self, in a person’s self-defined state of well-being [1]. This is much more complex than some biomedical approaches whereby the provider aims to ‘change’ behaviour [4]. Methods for this preliminary scanning have not yet been reported; to the best of our knowledge, no scoping review exists on this topic. The objective of this study will be to systematically map the literature on perspectives, concepts, and constructs of wellness and well-being in Indigenous communities in Canada.

In this paper, we describe a protocol for a scoping review on Indigenous perspectives of wellness and well-being in Canada.

This protocol will guide our scoping review which will inform the results from a larger funded study, a multi-site collaborative investigation of existing maternity service delivery models, and their impact on supporting persons to maintain health and wellness in the context of their own community [22]. Because our larger study is only focused on Northern Canadian communities, we have limited the parameters of this protocol to Indigenous people of Canada

While there is an array of review methodologies to use, we chose a scoping review for two distinct reasons. First, we are specifically interested in exploring conceptual boundaries related to Indigenous wellness to inform our current primary research study [1, 2]. Second, scoping reviews have matured as a methodologically rigorous approach [3–6] to ensure transparency and rigour in rapidly mapping a broad landscape of study designs and grey literature [1].

## Methods

### Study design

The review protocol has been registered within the Open Science Framework database (osf.io/3c5d4) and is being reported in accordance with the guidance provided in the Preferred Reporting Items for Systematic Reviews and Meta-analyses (PRISMA) extension for Scoping Reviews (PRISMA-ScR) [23] and the PRISMA extension for protocols (PRISMA-P) [24] (see PRISMA-P checklist in Additional file 1).

The Arksey and O’Malley 5-stage framework for conducting scoping reviews [25] will guide the methodology. Additionally, we will include a sixth step based on Levac et al.’s [26] recommendations as we did in an earlier scoping review [27].

The six steps will be as follows:

1. Develop a research question. In our study, our research questions of interest, in relation to Indigenous peoples in Canada are as follows:
  - How are well-being and wellness described in the literature, and what are the associated constructs?
  - What additional concepts are associated with well-being and/or wellness?
  - Are there indicators of well-being and/or wellness defined by Indigenous communities?
2. Search for relevant material.
3. Define study selection.
4. Chart the data.
5. Collate, summarize, and report results. Results will be organized in tables according to the research questions.
6. Consult with stakeholders. Results will be verbally shared for discussion with Indigenous elders via sharing circles.

We adopted Levac et al.’s stage 6 [26] to enhance the review through consultations with Indigenous stakeholders to share our results, and to compare and contrast with non-written concepts of wellness existing amongst communities.

### Eligibility criteria

The Population-Concept-Context (PCC) framework will be used to align the study selection with the research question [28]. While sources will not be limited by a specific time for review, they must include a focus on Indigenous peoples in Canada and measure or focus on specific concepts, perspectives, or constructs of well-being or wellness. Indigenous people in Canada include Inuit, Métis, and First Nations [29]. We acknowledge that some Indigenous peoples of Canada do not identify with these specific terms [23], but the terms are frequently used in Canadian documents.

Sources screened will include documents published in English, published and unpublished, including research of any study design, reviews and meta-analyses, dissertations, reports, books, and book chapters. Articles referencing Indigenous persons or communities residing on or off reserve as well as those living in urban settings will be included.

The literature included for review must describe or reflect Indigenous perspectives that identify concepts or constructs related to well-being or wellness; literature can be from any setting in Canada. Inclusion criteria in order of priority will be as follows:

1. Indigenous authored or co-authored. This will be determined as best as possible based on examples found in other published reviews [24, 30, 31] where Indigenous authorship is identified, our own knowledge of the scholars/writers, the writers’ own self-declarations, and any further inquiry we need to do.
2. Other authors, where the documents meet the other criteria listed here.
3. About Indigenous peoples (First Nations, Métis, Inuit) (e.g. amongst the interrelated peoples and cultures of Canada).
4. Take a strength-based approach; that is, assume that the perspectives described are affirming.
5. The literature must describe or reflect Indigenous perspectives regarding concepts or constructs of well-being or wellness. For example, the *First Nations Mental Wellness Continuum Framework* from Thunderbird Partnership Foundation [32] draws on established teachings. As the introduction describes, ‘It aims to support all individuals across the lifespan, including those with multiple and complex needs. The centre of the model refers to the interconnection between mental, physical, spiritual, and emotional behaviour—purpose, hope, meaning, and belonging. A balance between all of these elements leads to optimal mental wellness’ [32]. The framework and related documents apply to our scoping review because they were developed by community experts and knowledge keepers.

### Sources of evidence: search process

The literature will be retrieved through a structured search of electronic databases (from inception onwards): MEDLINE, EMBASE, Web of Science, CINAHL, Academic Search Complete, Anthropology Plus, Bibliography of Native North Americans, Canadian Business and Current Affairs, and Circumpolar Health Bibliographic Database.

The second source of relevant material will be retrieved through a search for grey literature (that is, not from peer-review journals) from websites (Additional file 2) and by using Google Scholar, Dissertations & Theses, iPortal Indigenous studies portal research tool, and Sociological Abstracts. The references of included documents will be followed up to identify any additional evidence sources, and a selected list of journals will be hand-searched. Scopus and Google Scholar will be used to identify citing literature.

The search strategy will be designed by one professional librarian (LD) and peer reviewed by an Indigenous health librarian (JL), using the Peer Review of Electronic Search Strategies (PRESS) checklist [33]. The input from the Indigenous scholar on our team (RS) will be integral for this step. The searches will be executed, and citations managed in a reference management system by the first librarian.

A preliminary scoping search of two databases, EMBASE for health literature and Canadian Business and Current Affairs for Canadian periodicals including relevant Indigenous and native study titles, will be conducted to identify all relevant keywords and subject headings. Following the initial search, the search strategies will be finalized and run in all identified resources. The initial search will be based on keywords describing Indigenous peoples in Canada and keywords describing the concepts of well-being and wellness. Database techniques including truncation, adjacency, and Boolean operators will be used as appropriate. A draft search strategy for EMBASE (Ovid) is provided in Additional file 3.

### Study selection

Based on the pre-defined inclusion criteria above, screening criteria will be established and piloted on a random 10% sample of abstracts and modified as necessary. Two researchers (KT and MHB) will screen the study title, authors, abstract, and descriptors for possible inclusion and full-text review. The same two investigators will independently review the full texts of potentially eligible studies for inclusion and resolve disagreements through discussion with additional reviewers (RS and PM) if necessary. The PRISMA flow diagram will be used to report all stages of the flow of the study selection [34].

### Data extraction

Data will be independently extracted by two investigators (KT and MHB) using a custom form that will be pilot tested. There will be two stages of data extraction, title and abstract review, and full article review. Disagreement will be resolved through discussion and additional reviewers (RS and PM) if necessary. Inter-rater agreement will be calculated with Cohen’s *K* statistic. Authors of the individual studies will be contacted for additional information if necessary.

### Descriptive variables

The following descriptive variables will be extracted for each included study, but additional categories may be identified iteratively as the literature is reviewed.

- Authors (Indigenous/non-Indigenous)
- Year of publication
- Jurisdictions or geographic location
- Nation
- Type of literature
- Language
- Concept(s) identified
- Construct(s) identified
- Articles acknowledged/considered the OCAP principles: ownership, control, access, and possession [35]
- Key findings on perspectives of well-being
- Key terminology used
- Source of perspectives (i.e. male, female, two-spirited, personal, community, reserve, rural, urban setting)

### Data management

Data will be managed with a shared spreadsheet file. The file will contain all the stages (title/abstract/full article review) of the review for both peer-reviewed literature and grey literature sources. All references will be organized in a shared folder amongst the team on Mendeley©. This ensures we account for duplicates and have accurate counts of each stage.

### Participants and public involvement

Our scoping review team has expertise in health and well-being, Indigenous scholarship and ways of knowing, and conducting scoping and systematic reviews. One of our team members is an Indigenous scholar who will provide insight and feedback throughout the project. Consultations with knowledge keepers and Indigenous stakeholders will be done in stage 6 of the scoping review to ensure no salient documents are missed.

### Data synthesis

The results of the review will be summarized with descriptive analysis (quantitative) and synthesis of the relevant information (context, concepts, constructs). We hope to create a meaningful diagram or table of wellness definitions and details around our findings. We will also engage Indigenous stakeholders to confirm and enhance the meaning of results from the review.

We will take our preliminary results to the larger community of elders and researchers who make up our grant team on the larger project. Their insights on the documents we retrieve and the knowledge they include will strengthen our interpretation and analysis. This step will be essential to ensuring we continue to honour the knowledge sharing that is the foundation of our work.

## Discussion

The scoping review in this protocol will identify, collect, and synthetize available literature on Indigenous perspectives, concepts, and constructs of wellness and well-being in Canada. We are not aware of another scoping review addressing this specific topic.

As we initiate a project on appropriate policy and systems approaches for maternal and child health and wellness in Indigenous communities, this scoping review will inform the language and paradigms to be considered and shared in the various sites and communities of our national project. In our opinion, this review will help to discuss relevant Indigenous constructs and concepts of wellness when we engage with Indigenous scholars, students, and the Northern Indigenous Advisory groups in the larger study to ensure the results are contextualized. Through the relationships we have cultivated in northern Indigenous communities, we have an opportunity to deepen our understanding of how Indigenous communities define wellness and well-being and engender discussions of how the review findings compare with unwritten knowledge of wellness and well-being.

The results of this review will also inform further stages of our grant, including the nature of questions asked for qualitative data collection, and can be used to inform new scholarship by other researchers in Canada. As a research team, we will determine an appropriate journal for publication

The potential limitations we anticipate based on the project thus far are that we may miss older documents and knowledge that are not available via library and online search strategies. The scoping review methodology will allow us to modify (and document any changes) the search protocols as needed. We do not anticipate any operational issues, as this research teams has worked well together in the past.

## Supplementary information

**Additional file 1.** PRISMA-P checklist.

**Additional file 2.** Identified websites relevant to Indigenous health and research in Canada.

**Additional file 3.** Proposed sample search strategy for EMBASE (Ovid).

## Data Availability

All data available from public sites and databases via the university.

## References

[1] Geddes. Measuring wellness: an indicator development guide for First Nations. 2015 [cited 2019 Aug 12]. Available from: https://static1.squarespace.com/static/558c624de4b0574c94d62a61/t/558f15c6e4b0c84f9abe4c66/1435440582698/BCFNDGI-Measuring-Wellness-An-Indicator-Development-Guide-for-First-Nations.pdf.

[2] Mitchell T, Maracle. Healing the generations: post-traumatic stress and the health status of Aboriginal populations in Canada. J Aborig Health. 2005;2.

[3] Adelson N (2005). The embodiment of inequity: health disparities in aboriginal Canada. Can J Public Heal Rev Can Santé Publique.

[4] Mirand AL, Beehler GP, Kuo CL, Mahoney MC (2002). Physician perceptions of primary prevention: qualitative base for the conceptual shaping of a practice intervention tool.

[5] Benoit C, Carroll D (1995). Aboriginal midwifery in British Columbia: a narrative untold. West Geogr Ser.

[6] Greenwood M, De Leeuw S, Lindsay NM, Reading C. Determinants of Indigenous peoples’ health: Canadian Scholars’ Press; 2015.

[7] Davis B. Cultural connectedness as personal wellness in First Nations youth. Western University; 2012 [cited 2019 Jun 6]. Available from: https://ir.lib.uwo.ca/etd/403/.

[8] Riecken T, Scott T, Tanaka M. Community and culture as foundations for resilience: participatory health research with First Nations student filmmakers. J Aborig Health. 2006 [cited 2019 Jun 6];3(1):7–14. Available from: https://jps.library.utoronto.ca/index.php/ijih/article/view/28950.

[9] Big-Canoe K, Richmond C. Anishinabe youth perceptions about community health: toward environmental repossession. Health Place. 2014 [cited 2019 Jun 6];26:127–35. Available from: https://www.sciencedirect.com/science/article/pii/S1353829213001779. 10.1016/j.healthplace.2013.12.013 24440804

[10] Gray A, Richer F, Health SH-C Journal of Public, 2016 U. Individual-and community-level determinants of Inuit youth mental wellness. Can J Public Heal. 2016 [cited 2019 Jun 6];107(3):e251–7. Available from: http://link.springer.com/10.17269/CJPH.107.5342. 10.17269/CJPH.107.5342 PMC6972064 27763839

[11] Isaak CA, Marchessault G (2008). Meaning of health: the perspectives of Aboriginal adults and youth in a Northern Manitoba First Nations community. Can J Diabetes.

[12] Barker B, Goodman A, De Beck K. Reclaiming Indigenous identities: culture as strength against suicide among Indigenous youth in Canada. Can J Public Heal. 2017 [cited 2019 Jun 6];108(2):208–10. Available from: https://link.springer.com/article/10.17269/CJPH.108.5754. 10.17269/CJPH.108.5754 PMC6972180 28621659

[13] Victor J, Linds W, Episkenew J-A, Goulet L, Benjoe D, Brass D (2016). Kiskenimisowin (self-knowledge): co-researching wellbeing with Canadian First Nations youth through participatory visual methods. Int J Indig Heal.

[14] Warne D. Traditional perspectives on child and family health. Paediatr Child Health. 2005 [cited 2019 Jun 6];10(9):542–4. Available from: https://academic.oup.com/pch/article-abstract/10/9/542/2648379. 10.1093/pch/10.9.542 PMC2722639 19668686

[15] Wexler L. The importance of identity, history, and culture in the wellbeing of Indigenous youth. J Hist Child Youth. 2009 [cited 2019 Jun 6];2(2):267–76. Available from: https://muse.jhu.edu/article/266442/summary.

[16] Greenwood M (2005). Children as citizens of First Nations: linking Indigenous health to early childhood development. Paediatr Child Health (Oxford).

[17] Ordolis E. A Story of their own: adolescent pregnancy and child welfare in Aboriginal communities. First Peoples Child Fam Rev. 2007 [cited 2019 Jun 6];3(4):30–41. Available from: https://pdfs.semanticscholar.org/68ed/c1e5e423b865640586bbacb55f42823302fb.pdf.

[18] Pigford AA, Willows ND, Holt NL, Newton AS, Ball GD (2012). Using First Nations children’s perceptions of food and activity to inform an obesity prevention strategy. Qual Health Res.

[19] Hatala A, Morton D, Njeze C, Bird-Naytowhow K, Pearl T. Re-imagining miyo-wicehtowin: human-nature relations, land-making, and wellness among Indigenous youth in a Canadian urban context. Soc Sci Med. 2019;230:122–30 Available from: https://www-sciencedirect-com.uml.idm.oclc.org/science/article/pii/S0277953619302126. 10.1016/j.socscimed.2019.04.012 31009878

[20] Lines L, Yellowknives Dene First Nation Wellness Division, Jardine C. Connection to the land as a youth-identified social determinant of Indigenous peoples’ health. BMC Public Health. 2019 [cited 2019 Jun 6];19(1):1–13. Available from: http://www.ncbi.nlm.nih.gov/pubmed/30744592. 10.1186/s12889-018-6383-8 PMC6371607 30744592

[21] Snowshoe A, Crooks C, Tremblay P, Hinson R. Cultural connectedness and its relation to mental wellness for First Nations youth. J Prim Prev. 2017 [cited 2019 Jun 6];38(1):67–86. Available from: http://www.ncbi.nlm.nih.gov/pubmed/27807659. 10.1007/s10935-016-0454-3 27807659

[22] Thiessen K, Whitecloud K (2018). Welcoming the “sacred spirit” (child): connecting Indigenous and Western “ways of knowing” to inform future policy partnership to optimize maternal child health service delivery initiatives in remote Canadian regions.

[23] Aboriginal Identity & Terminology. [cited 2020 Jun 30]. Available from: https://indigenousfoundations.arts.ubc.ca/aboriginal_identity__terminology/.

[24] Stefanelli RD, Castleden H, Harper SL, Martin D, Cunsolo A, Hart C (2017). Experiences with integrative Indigenous and Western knowledge in water research and management: a systematic realist review of literature from Canada, Australia, New Zealand, and the United States. Environ Rev.

[25] Arksey H, O’Malley L. Scoping studies: towards a methodological framework. Int J Soc Res Methodol. 2005 Feb [cited 2018 Jul 2];8(1):19–32. Available from: http://www.tandfonline.com/doi/abs/10.1080/1364557032000119616.

[26] Levac D, Colquhoun H, O’Brien KK. Scoping studies: advancing the methodology. Implement Sci. 2010 20 [cited 2018 Jul 2];5(1):69. Available from: http://implementationscience.biomedcentral.com/articles/10.1186/1748-5908-5-69. 10.1186/1748-5908-5-69 PMC2954944 20854677

[27] Thiessen K, Haworth-Brockman M, Nurmi MA, Demczuk L, Sibley KM (2020). Delivering midwifery: a scoping review of employment models in Canada. J Obstet Gynaecol Can.

[28] Peters MD, Godfrey CM, McInerney P, Soares CB, Khalil H, Parker D. The Joanna Briggs Institute Reviewers’. Manual 2015: Methodology for JBI scoping reviews. Joanne Briggs Inst. 2015:1–24 Available from: http://joannabriggs.org/assets/docs/sumari/ReviewersManual_Mixed-Methods-Review-Methods-2014-ch1.pdf.

[29] Indigenous peoples and cultures - Canada.ca. [cited 2020 Jun 30]. Available from: https://www.canada.ca/en/services/culture/canadian-identity-society/indigenous-peoples-cultures.html.

[30] Jones J, Cunsolo A, Harper SL (2018). Who is research serving? A systematic realist review of circumpolar environment-related Indigenous health literature. PLoS One.

[31] Hutchings K, Bainbridge R, Bodle K, Miller A (2019). Determinants of attraction, retention and completion for Aboriginal and Torres Strait Islander higher degree research students: a systematic review to inform future research directions. Res High Educ.

[32] First Nations Mental Wellness Continuum Framework | Thunderbird Partnership Foundation. [cited 2020 Jun 30]. Available from: https://thunderbirdpf.org/first-nations-mental-wellness-continuum-framework/.

[33] Mcgowan J, Sampson M, Salzwedel D, Cogo E, Foerster V, Lefebvre C (2016). PRESS peer review of electronic search strategies: 2015 guideline statement. J Clin Epidemiol.

[34] Stovold E, Beecher D, Foxlee R, Noel-Storr A. Study flow diagrams in Cochrane systematic review updates: an adapted PRISMA flow diagram. Syst Rev. 2014;3:54. 10.1186/2046-4053-3-54 PMC4046496 24886533

[35] OCAP® | FNIGC. [cited 2020 Jun 30]. Available from: https://fnigc.ca/ocap.

