# Supplementary material for: Indigenous perspectives on wellness and health in Canada: study protocol for a scoping review
Source: Syst Rev. 2020 Aug 11;9:177. doi: 10.1186/s13643-020-01428-0 (PMC7418305; doi:10.1186/s13643-020-01428-0)
Supplement: Supplementary file 2 — Additional file 2. Identified websites relevant to Indigenous health and research in Canada. [file 13643_2020_1428_MOESM2_ESM.docx]

Additional File 2: Identified websites relevant to Indigenous health and research in Canada

1. National Collaborating Centre for Indigenous Health
2. Native Women’s Association of Canada
3. Assembly of First Nations
4. Pauktuutit (Inuit Women’s Association)
5. Inuit Tapiri Kanatami
6. Metis Nation of Canada
7. Canadian Indigenous Nurses Association
8. First Nations Information Governance Centre
9. Aboriginal Peoples’ Survey
10. Canadian Institute of Child Health
11. Statistics Canada
12. Best Start Resource Centre
13. First Nations Health Authority
14. National Aboriginal Midwives Association
15. British Columbia Aboriginal Child Care Society
16. Canadian Paediatric Society
17. Better Beginnings, Better Futures
18. First Nations Child and Family Caring Society of Canada
