# Supplementary material for: Indigenous perspectives on wellness and health in Canada: study protocol for a scoping review
Source: Syst Rev. 2020 Aug 11;9:177. doi: 10.1186/s13643-020-01428-0 (PMC7418305; doi:10.1186/s13643-020-01428-0)
Supplement: Supplementary file 3 — Additional file 3. Proposed sample search strategy for EMBASE (Ovid). [file 13643_2020_1428_MOESM3_ESM.docx]

Additional File 3 Proposed Sample search strategy for EMBASE (Ovid)

| 1 | Indigenous people/ or First Nation/ or American Indian/ or Metis/ or Eskimo/ or Inuit/ |
| --- | --- |
| 2 | (Algonquin or Anishina* or Anishna* or Athapaskan or Athabaskan or Atikamekweet or Blackfoot or Cayuga or Chipewyan or Cree or Dene or Dakelh or Dunne-za or Gitskan or Gitsxan or Gwich’in or Haida or Haisla or Haudenosaunee or Heiltsuk or Huron or Iroquois or Kaska or Ktunaxa or Kwakwaka'wakw or Malis or Mi'kmaq or Micmac or Mohawk or Nakoda or Nipissing or Nisga'a or Nlaka'pamux or Nuu chah nulth or Nuxalk or Ojibw* or Oji-Cree or Okanagan or Oneida or Onondaga or Oweenkeno or Passamaquoddy or Potawatomi or Salish or Sec wepmc or Seneca or Six Nations or Saulteaux or Sekani or Stl'atlimc or Tagish or Tahltan or Tasttine or Tlingit or Tsilhqot'in or Tsimshian or Tsuu T'inia or Tuscarora or Tutchone or Wakashan or Wet'suwet'en or Wyandot).mp. |
| 3 | Aboriginal* or Indigenous or Metis or First Nation or First Nations or Amerindian* or “on reserve” or off-reserve or Autochtone* or Inuit* or Innu or Montagnais or Inuk or Inuvialuit* or Inuktitut or Eskimo*.mp. |
| 4 | (urban adj3 (Indian* or Native* or Aboriginal*)).mp. |
| 5 | (Native* adj1 (American or man or men or women or woman or mother* or grandmother* or father* or grandfather* or elder* or child* or baby or babies or infant or infants or boy* or girl* or adolescent* or youth or youths or teen* or person* or adult* or people* or Indian* or Nation or tribe* or tribal or band or bands)).mp. |
| 6 | 1 or 2 or 3 or 4 or 5 |
| 7 | Canadian Aboriginal/ |
| 8 | Canad* adj1 (Native* or Indian*).mp. |
| 9 | 7 or 8 |
| 10 | Exp Canada/ |
| 11 | (Canad* or British Columbia or Columbie Britannique or Alberta or Saskatchewan or Manitoba or Ontario or Quebec or Nova Scotia or Nouvelle Ecosse or New Brunswick or Nouveau Brunswick or Newfoundland or Terre Neuve or Labrador or Prince Edward Island or Yukon or NWT or Northwest Territories or Yellowknife or Whitehorse or Nunavut or Nunavummiut or Nunavik or Nunatsiavut or NunatuKavut OR Nunangat OR Nunatsiavut OR Nitassinan or Inuvialuit OR Kuujjuaq OR Qaujigiartilt or Kitikmeot or Kivalliq or Qikiqtaaluk or Qikiqtani).mp |
| 12 | 10 or 11 |
| 13 | 6 and 12 |
| 14 | 9 or 13 |
| 15 | wellbeing/ or physical well-being/ or psychological well-being/ |
| 16 | well-being or well being or wellbeing or wellness.mp |
| 17 | (heal or healing* or healer* or resilien* or holistic or wholistic or wholeness or balance or harmony).mp. |
| 18 | 15 or 16 or 17 |
| 19 | 14 and 18 |
| 20 | “ways of knowing”.mp. |
| 21 | traditional medicine/ or traditional healer/ |
| 22 | Health* adj6 (perspective* or meaning or meanings or concept* or perception* or belief* or knowledge or knowing or worldview* or view* or value* or epistemolog* or paradigm* or lens or seeing or teaching*).mp. |
| 23 | 20 or 21 or 22 |
| 24 | 14 and 23 |
| 25 | 19 or 24 |

Notes:

term/ indicates subject heading

exp term/ indicates exploded subject heading

* indicates truncation

adj indicates adjacency operator
